# Supplementary material for: Anaemia and associated factors in people living with HIV on ART in Southern Province of Zambia
Source: PLoS One. 2025 Sep 23;20(9):e0331643. doi: 10.1371/journal.pone.0331643 (PMC12456802; doi:10.1371/journal.pone.0331643)
Supplement: Attachment — Submitted filename: PONE-D-25-24917 REVIEW.docx [file pone.0331643.s003.docx]

**Manuscript Number and title: PONE-D-25-24917 Anaemia and associated factors in people living with HIV on ART in Southern Province of Zambia**

The article reports a retrospective cohort study in which anaemia prevalence, sex differences and associated factors were determined among 2,840 PLHIV aged ≥15 years from 12 districts in Southern Zambia, using secondary data collected from medical records. Anaemia prevalence among the cohort was determined as 15.7%, with prevalence being higher among the female participants. Cotrimoxazole (CTX) prophylaxis reduced the likelihood of anaemia. Likewise, participants on INSTI-based ART had a lower anaemia prevalence in comparison to other regimens. Viral load was independently associated with anaemia (AOR: 1.00, p=0.021) and advanced WHO stages (Stage 3: 50% anaemia) and lower creatinine levels correlated with increased risk. Based on these findings, the authors concluded that gender-responsive interventions are necessary in order to deal with the significant sex-based disparities in anaemia which was observed. They also underscored the importance of broader integration of INSTI-based ART regimens and CTX prophylaxis into HIV care, as a means of anaemia management in high burden settings.

The problem of hematological abnormalities such as anaemia among persons living with HIV is well established. However, this current study makes a relevant contribution to the literature on the predictors of anaemia among PLHIV in Sub-Saharan Africa.

**Issues identified**

1. The objectives outlined in the introduction on page 4 lines 78-80 *(“This study evaluates how combined strategies routine haemoglobin checks, gender-responsive nutrition support, and infection-specific treatment can reduce anaemia-related illness and deaths among people with HIV on antiretroviral therapy in Sub-Saharan Africa”*) should be rewritten to align with those in the abstract (Page 2 line 27-29). It is a better reflection of what the study set out to do.
2. Rephrase lines 81-83 (page 4). The study findings clearly highlight gender-based disparities which must be addressed in order to improve HIV care and lessen the burden of anaemia. However, addressing these disparities per se were not within the scope of the study. In view of this the remarks on page 4 lines 81 and 83 “*By addressing gender-based disparities and systemic health inequities, it* [i.e. the study] *aims to create scalable solutions to improve HIV care, advance global HIV goals, and lessen the dual burden of anaemia and inequality in high-prevalence regions*” should be rephrased.
3. Page 6 line 149- reference should be made to Table 1 and not Table 2.
4. Page 9 line 177-178. The title for Table 2 includes the phrase, ‘…haemoglobin concentration at 12 months’ however it is not immediately clear what the 12 months is/signifies. A brief explanation can be included as a footnote to the table.
5. Page 10 line 190. Please check reference 20. It is unclear what information was cited from it. The citation may be erroneous.
