# Supplementary material for: Anaemia and associated factors in people living with HIV on ART in Southern Province of Zambia
Source: PLoS One. 2025 Sep 23;20(9):e0331643. doi: 10.1371/journal.pone.0331643 (PMC12456802; doi:10.1371/journal.pone.0331643)
Supplement: Attachment — Submitted filename: Response_to_Reviewers[1].docx [file pone.0331643.s005.docx]

10/07/2025

PLOS ONE Journal

Dear Editor,

**Ref: Submission of a revised research article for peer review and publication consideration**

Reference to the above-mentioned subject. I am writing to submit a revised original research article titled "Anaemia and associated factors in people living with HIV on ART in Southern Province of Zambia,".

We would like to thank the reviewers for taking the time to make suggestions that have improved our manuscript. We have revised the manuscript and addressed all concerns and suggestions. We now hope the current manuscript is acceptable for publication. Below are the point-by-point responses to all comments and suggestions.

**Response to the reviewer: No. 1**

The objectives outlined in the introduction on page 4 lines 78-80 *(“This study evaluates how combined strategies routine haemoglobin checks, gender-responsive nutrition support, and infection-specific treatment can reduce anaemia-related illness and deaths among people with HIV on antiretroviral therapy in Sub-Saharan Africa”*) should be rewritten to align with those in the abstract (Page 2 line 27-29). It is a better reflection of what the study set out to do.

**Response**: Thank you very much for the suggestion: we have revised the manuscript the manuscript as suggested.

**Reviewer comments: No. 2**

Rephrase lines 81-83 (page 4). The study findings clearly highlight gender-based disparities which must be addressed in order to improve HIV care and lessen the burden of anaemia. However, addressing these disparities per se were not within the scope of the study. In view of this the remarks on page 4 lines 81 and 83 “*By addressing gender-based disparities and systemic health inequities, it* [i.e. the study] *aims to create scalable solutions to improve HIV care, advance global HIV goals, and lessen the dual burden of anaemia and inequality in high-prevalence regions*” should be rephrased.

**Response:** We have revised the manuscript the manuscript as suggested. We included a table (table 2) showing gender based disparities.

**Reviewer comments: No. 3**

Page 6 line 149- reference should be made to Table 1 and not Table 2.

**Response**: Thank you very much for the suggestion: we have revised the manuscript the manuscript as suggested.

**Reviewer comments: No. 4**

Page 9 line 177-178. The title for Table 2 includes the phrase, ‘…haemoglobin concentration at 12 months’ however it is not immediately clear what the 12 months is/signifies. A brief explanation can be included as a footnote to the table.

**Response**: Thank you very much for the suggestion: we have revised the manuscript the manuscript as suggested. We removed the contradicting statement which was 12 months

**Reviewer comments: No. 5**

Page 10 line 190. Please check reference 20. It is unclear what information was cited from it. The citation may be erroneous.

**Response**: Thank you very much for the suggestion: we have revised the manuscript the manuscript as suggested. The reference was worked on.

We have revised the manuscript and addressed all concerns raised. We want to thank you all again for the tremendous work and time that you committed to reviewing and correcting our work. Our manuscript is much improved, and we are very grateful.

Please address all correspondence to [chakulyamartin1@gmail.com](mailto:chakulyamartin1@gmail.com) . We look forward to hearing from you at your earliest convenience.

Please do not hesitate to contact me should you have further questions.

Yours sincerely,

Mr Chakulya Martin, BSc., MSc.

Lecturer Mulungushi University Teaching Hospital
